# Brain-specific transcriptional regulator T-brain-1 controls brain wiring and neuronal activity in autism spectrum disorders

**DOI:** 10.3389/fnins.2015.00406

**Published:** 2015-11-03

**Authors:** Tzyy-Nan Huang, Yi-Ping Hsueh

**Affiliations:** Institute of Molecular Biology, Academia SinicaTaipei, Taiwan

**Keywords:** amygdala, axonal projection, autism, cerebral cortex, intellectual disability, neural circuit, neurodevelopmental disorders, TBR1

## Abstract

*T-brain-1* (*TBR1*) is a brain-specific T-box transcription factor. In 1995, *Tbr1* was first identified from a subtractive hybridization that compared mouse embryonic and adult telencephalons. Previous studies of *Tbr1*^−∕−^ mice have indicated critical roles for TBR1 in the development of the cerebral cortex, amygdala, and olfactory bulb. Neuronal migration and axonal projection are two important developmental features controlled by TBR1. Recently, recurrent *de novo* disruptive mutations in the *TBR1* gene have been found in patients with autism spectrum disorders (ASDs). Human genetic studies have identified *TBR1* as a high-confidence risk factor for ASDs. Because only one allele of the TBR1 gene is mutated in these patients, *Tbr1*^+∕−^ mice serve as a good genetic mouse model to explore the mechanism by which *de novo TBR1* mutation leads to ASDs. Although neuronal migration and axonal projection defects of cerebral cortex are the most prominent phenotypes in *Tbr1*^−∕−^ mice, these features are not found in *Tbr1*^+∕−^ mice. Instead, inter- and intra-amygdalar axonal projections and NMDAR expression and activity in amygdala are particularly susceptible to *Tbr1* haploinsufficiency. The studies indicated that both abnormal brain wiring (abnormal amygdalar connections) and excitation/inhibition imbalance (NMDAR hypoactivity), two prominent models for ASD etiology, are present in *Tbr1*^+∕−^ mice. Moreover, calcium/calmodulin-dependent serine protein kinase (CASK) was found to interact with TBR1. The CASK–TBR1 complex had been shown to directly bind the promoter of the *Grin2b* gene, which is also known as *Nmdar2b*, and upregulate *Grin2b* expression. This molecular function of TBR1 provides an explanation for NMDAR hypoactivity in *Tbr1*^+∕−^ mice. In addition to *Grin2b*, cell adhesion molecules—including *Ntng1, Cdh8*, and *Cntn2*—are also regulated by TBR1 to control axonal projections of amygdala. Taken together, the studies of *Tbr1* provide an integrated picture of ASD etiology at the cellular and circuit levels.

## Introduction

Autism spectrum disorders (ASDs) are heterogeneous and highly heritable neuropsychiatric disorders. Hundreds of genes with *de novo* copy-number variations or *de novo* point mutations have been identified in thousands of patients with ASDs (Gilman et al., 2011; Neale et al., 2012; O'roak et al., 2012a,b; De Rubeis et al., 2014; Iossifov et al., 2014). Although this variety of ASD-associated genes reflects the high heterogeneity of ASDs, ~26 high-confidence risk genes for ASDs have been summarized from large scale whole-exome sequencing (O'roak et al., 2012a; De Rubeis et al., 2014; Table 1). Among these high-confidence risk genes, 11 encode either transcription factors or chromatin remodeling factors, indicating that the dysregulation of gene expression is a common pathogenic mechanism for ASDs (Table 1). To date, *T-BRAIN-1* (*TBR-1*) is the best studied transcription regulator among the high-confidence risk genes for ASDs. In this review, we summarize the physiological functions of TBR1 and the currently understood mechanisms by which *TBR1* mutations cause ASDs. Based on the data accumulated from the mouse model, we suggest that abnormal brain wiring and reduced neuronal activity in the amygdala are the primary causes for *TBR1*-dependent ASDs.

**Table 1:** **High-confidence risk factors for ASDs**.

**Gene symbol**	**Gene Name**	**Molecular function**	**References**
ADNP	Activity-dependent neuroprotector homeobox	Transcription regulator	De Rubeis et al., 2014; SFARI
ANK2	Ankyrin 2, neuronal	Cytoskeleton interactor	De Rubeis et al., 2014; SFARI
ARID1B	AT rich interactive domain 1B (SWI1-like)	Transcription regulator	De Rubeis et al., 2014; SFARI
ASH1L	Ash1 (absent, small, or homeotic)-like (Drosophila)	Transcription regulator	De Rubeis et al., 2014; SFARI
ASXL3	Additional sex combs like transcriptional regulator 3	Transcription regulator	De Rubeis et al., 2014; SFARI
BCL11A	B-cell CLL/lymphoma 11A (zinc finger protein)	Transcription regulator	De Rubeis et al., 2014
CACNA2D3	Calcium channel, voltage-dependent, alpha 2/delta subunit 3	Ion channel	De Rubeis et al., 2014
CHD8	Chromodomain helicase DNA binding protein 8	Transcription regulator	De Rubeis et al., 2014; O'roak et al., 2012a; SFARI
CTTNBP2	Cortactin binding protein 2	Cytoskeleton interactor	De Rubeis et al., 2014
CUL3	Cullin 3	Protein degradation	De Rubeis et al., 2014
DYRK1A	Dual-specificity tyrosine-(Y)-phosphorylation regulated kinase 1A	Signaling	De Rubeis et al., 2014; O'roak et al., 2012a; SFARI
GABRB3	Gamma-aminobutyric acid (GABA) A receptor, beta 3	Ion channel	De Rubeis et al., 2014
GRIN2B	Glutamate receptor, ionotropic, N-methyl D-aspartate 2B	Ion channel	De Rubeis et al., 2014; O'roak et al., 2012a; SFARI
KATNAL2	Katanin p60 subunit A-like 2	Cytoskeleton interactor	De Rubeis et al., 2014
MIB1	Mindbomb E3 ubiquitin protein ligase 1	Protein degradation	De Rubeis et al., 2014
MLL3	Lysine (K)-specific methyltransferase 2C	Transcription regulator	De Rubeis et al., 2014
POGZ	Pogo transposable element with ZNF domain	Enzyme	De Rubeis et al., 2014; SFARI
PTEN	Phosphatase and tensin homolog	Phosphatase	De Rubeis et al., 2014; O'roak et al., 2012a; SFARI
RELN	Reelin	Signaling	De Rubeis et al., 2014
SCN2A	Sodium channel, voltage-gated, type II, alpha subunit	Ion channel	De Rubeis et al., 2014; SFARI
SETD5	SET domain containing 5	Transcription regulator	De Rubeis et al., 2014; SFARI
SHANK3	SH3 and multiple ankyrin repeat domains 3	Postsynaptic adaptor	SFARI
SUV420H1	Suppressor of variegation 4–20 homolog 1 (Drosophila)	Transcription regulator	De Rubeis et al., 2014; SFARI
SYNGAP1	Synaptic Ras GTPase activating protein 1	Signaling	De Rubeis et al., 2014; SFARI
TBL1XR1	Transducin (beta)-like 1 X-linked receptor 1	Transcription regulator	O'roak et al., 2012a
TBR1	T-box, brain, 1	Transcription regulator	De Rubeis et al., 2014; O'roak et al., 2012a; SFARI

## Identification of TBR1 in the regulation of brain development

**Figure 1:**
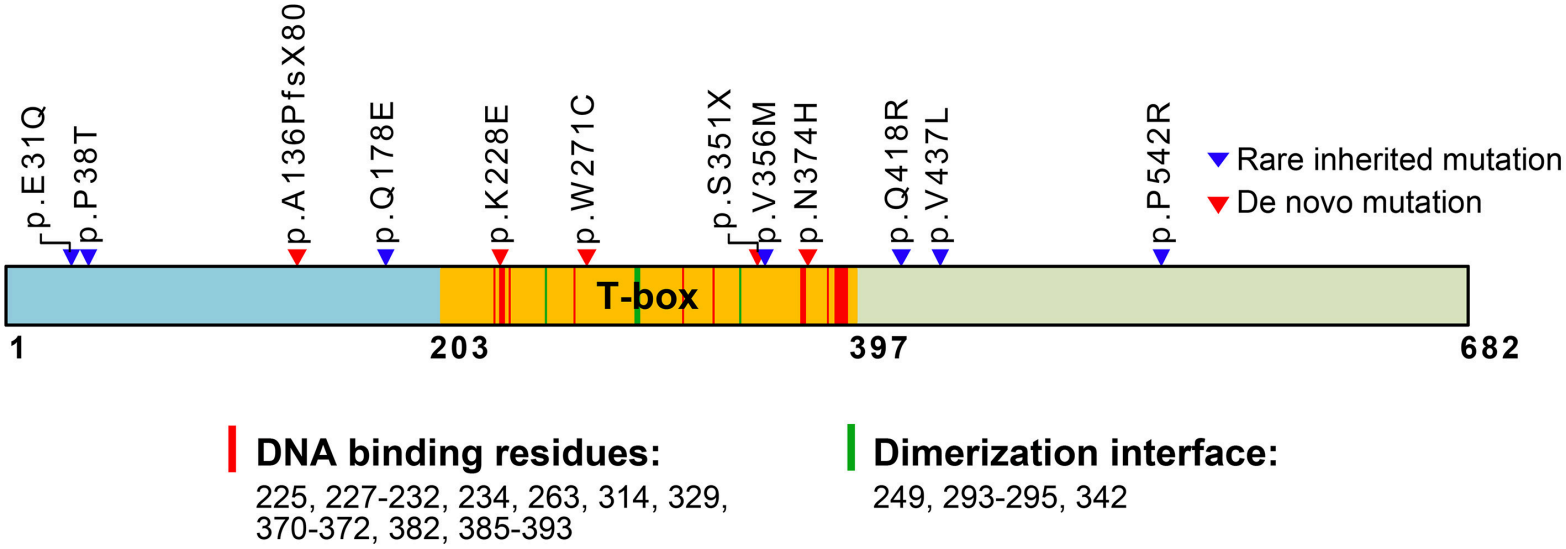
**Schematic domain structure of TBR1 and identified mutations in patients with ASDs**. The T-box DNA binding domain extends from amino acid (aa) residue 203–397. The predicted aa residues for DNA binding and dimerization based on the T-box structure of Brachyury (T protein) are also indicated and labeled with red and green strips in the T-box. The positions of *de novo* mutations are labeled with red triangles; the positions of rare inherited mutations are labeled with blue triangles. The functions of the residues in the T-box are predicted based on comparison with the Brachury T-box (pfam00907: T-box, http://www.ncbi.nlm.nih.gov/Structure/cdd/cddsrv.cgi?uid=250216).

TBR1 contains a T-box DNA binding domain (Figure 1) and belongs to the T-box transcription factor family (Papaioannou, 2014). Twenty years ago, Dr. John Rubenstein's laboratory first identified *Tbr1* from a subtractive hybridization screen using cDNA libraries made from mouse embryonic day 14.5 (E14.5) and adult telencephalons (Bulfone et al., 1995). *Tbr1* mRNA levels were approximately 10-fold higher in E14.5 telencephalons than in adult telencephalons (Bulfone et al., 1995), suggesting a role for TBR1 in brain development. *In situ* hybridization and immunofluorescence staining indicate that *Tbr1* is expressed in the postmitotic neurons of the cerebral cortex, hippocampus, olfactory bulb and amygdala at the embryonic stages (Bulfone et al., 1995, 1998; Remedios et al., 2007; Huang et al., 2014). Using markers of projection neurons, including glutamate and CaMKII, TBR1 has been found to be further restricted to the projection neurons of the cerebral cortex, amygdala and olfactory bulb (Bulfone et al., 1998; Hevner et al., 2001; Huang et al., 2014). In the cerebral cortex, layer 6 neurons express the highest levels of TBR1. Projection neurons in the remaining layers also express TBR1, though the expression levels are lower (Hevner et al., 2001). In the amygdala, TBR1 is only expressed in the projection neurons of the lateral and basal amygdala (Huang et al., 2014). These studies clearly show that TBR1 is a projection neuron-specific T-box factor highly enriched in embryonic telencephalons.

Dr. John Rubenstein and colleagues generated *Tbr1*^−∕−^ mice to investigate the physiological function of *Tbr1* (Bulfone et al., 1998; Hevner et al., 2001). A homozygous deficiency of *Tbr1* results in neonatal lethality within 1–2 days after birth, indicating that *Tbr1* is essential for survival. Most projection neurons in the olfactory bulb, including mitral and tufted cells, and axonal output to the lateral olfactory tract are lost in *Tbr1*^−∕−^ mice (Bulfone et al., 1998). In the cerebral cortex, the inside-out pattern of neuronal migration is completely disrupted, as the six-layer laminar structure of the cortex is disorganized. Moreover, the contralateral axonal projections of the cerebral cortex and both corticothalamic and thalamocortical axonal projections are also defective, since they end mid-way to their final destinations in *Tbr1*^−∕−^ mice (Hevner et al., 2001). TBR1 is also required for neuronal migration in the amygdala. A portion of the dorsal pallium that migrates from the caudal telencephalon pole toward the rostral telencephalon forms the basal and lateral amygdala. In *Tbr1*^−∕−^ mice, this caudal-to-rostral migration is disrupted and thus impairs amygdala development (Remedios et al., 2007). Although TBR1 is also expressed in the hippocampus, its importance in hippocampus development and function remains unclear.

Based on the studies using *Tbr1*^−∕−^ mice, it is clear that TBR1 is critical for development of projection neurons in the cerebral cortex, olfactory bulb and amygdala.

## TBR1 downstream target genes

Target genes of the TBR1 transcription factor were first identified by searching a database using the target sequence of the T-box DNA binding domain (Hsueh et al., 2000; Wang et al., 2004a,c). Because members of the T-box protein family share a DNA binding sequence, this method cannot ensure that the target genes are specific for TBR1. Because TBR1 is neuron-specific, neuronal expression is the first criterion to further screen the TBR1 target genes identified from sequence analysis. Results of an electrophoretic mobility shift assay, chromatin immunoprecipitation and a luciferase reporter assay have shown that TBR1 directly binds to the promoters and regulates the promoter activity of *Grin2b* (*Glutamate receptor, ionotropic, N-methyl-aspartate 2b*, also known as *Nmdar2b*) and *Reln* (*Reelin*) (Hsueh et al., 2000; Wang et al., 2004a,c). Changes in RELN and NMDAR2B protein levels have also been confirmed in *Tbr1*^−∕−^ mice (Hevner et al., 2001; Wang et al., 2004c). Because *Reln* encodes an extracellular protein that is critical for neuronal migration (Martinez-Cerdeno and Noctor, 2014; Ohshima, 2014; Sekine et al., 2014), regulation of *Reln* expression by TBR1 could explain the migration phenotype in *Tbr1*^−∕−^ mice. Regulation of *Grin2b* expression by TBR1 is critical for neuronal activation, which we discuss further in a later section.

Both our and Dr. Robert Hevner's laboratories independently applied microarray analyses to identify TBR1 downstream genes. Using E14.5 and P0.5 mouse brains, Dr. Hevner's laboratory focused on the arealization and lamination of the cerebral cortex (Bedogni et al., 2010). *Tbr1* exhibits a high rostral and low caudal expression pattern in the cortex (Bulfone et al., 1995). At both E14.5 and P0.5, a *Tbr1* deletion noticeably alters the expression of regional markers. In general, rostral genes are downregulated in *Tbr1*^−∕−^ brains, while caudal genes are upregulated. For cortical layer markers, most markers of layer 6, subplate and Cajal–Retzius cells exhibit noticeably reduced expression levels in *Tbr1*^−∕−^ brains. The majority of layer 2–5 markers are upregulated (Bedogni et al., 2010). The markers of lamination and arealization whose expression levels are altered in *Tbr1*^−∕−^ brains are listed in Table 2. These studies indicate that TBR1 is critical for controlling the neuronal specification of the cerebral cortex.

**Table 2:** *****Tbr1*** deletion alters expression of genes with layer- or region-specific distribution in the cerebral cortex**.

Gene symbol^*^	**Gene name**	**Layer**	**Region**
Bhlhb5 (BHLHE22)	Basic helix-loop-helix family, member e22	L5	Caudomedial
Calb2	Calbindin 2	Cajal–Retzius	All
Cdh8^*^	Cadherin 8, type 2	L5	Rostral + caudal
Cdh9	Cadherin 9, type 2 (T1-cadherin)	L6	Rostral
Cntn3	Contactin 3 (plasmacytoma associated)	L5	Caudal
Cntn6^*^	Contactin 6	L5 SCPN	Caudal
Cplx3	Complexin 3	Cajal–Retzius	All
Crim1^*^	Cysteine rich transmembrane BMP regulator 1 (chordin-like)	L5 corticospinal motor neuron	All
Crym	Crystallin, mu	L5 corticospinal motor neuron	Caudal
Ctgf^*^	Connective tissue growth factor	Subplate	All
Ctip2 (Bcl11b)	B-cell CLL/lymphoma 11B (zinc finger protein)	L5 SCPN	All
Cux1	Cut-like homeobox 1	L2–4	All
Cux2	Cut-like homeobox 2	L2–4	All
Dkk3	Dickkopf WNT signaling pathway inhibitor 3	CPN	Caudomedial
Drd1a^*^	Dopamine receptor D1	L6	All
Dtx4	Deltex 4, E3 ubiquitin ligase	L2–4	All
Etv1 (Er81)	Ets variant 1	L5	All
Fezf2	FEZ family zinc finger 2	L5 SCPN	All
Flrt3^*^	Fibronectin leucine rich transmembrane protein 3	L2–3	Caudal
Foxp1	Forkhead box P1	L5	All
Foxp2	Forkhead box P2	L6	All
Inhba	Inhibin, beta A	L2–4, CPN	All
Lhx5	LIM homeobox 5	Cajal–Retzius	All
Limch1	LIM and calponin homology domains 1	L2–3, CPN	All
Mdga1	MAM domain containing glycosylphosphatidylinositol anchor 1	L2–3	Caudal
Mef2c	Myocyte enhancer factor 2C	L2–3	All
Nefm^*^	Neurofilament, medium polypeptide	L2–3	Caudal
Nfe2l3	Nuclear factor, erythroid 2-like 3	L6	All
Ngfr	Nerve growth factor receptor	L6	Caudal
Nhlh2^*^	Nescient helix loop helix 2	Cajal–Retzius	All
Npy	Neuropeptide Y	L6	All
Nr4a2 (Nurr1)^*^	Nuclear receptor subfamily 4, group A, member 2	L6	Caudal
Nr4a3	Nuclear receptor subfamily 4, group A, member 3	L6	Caudal
Nrgn^*^	Neurogranin	L2–4	All
Nxph4	Neurexophilin 4	Subplate	All
Oma1	OMA1 zinc metallopeptidase	L5 SCPN	All
Pcdh11x	Protocadherin 11 X-linked	L2–4	All
Pcdh19	Protocadherin 19	L5	All
Pcdh20	Protocadherin 20	L2–4	Rostral
Pcdh8	Protocadherin 8	L2–3	Caudal
Pcp4	Purkinje cell protein 4	L5	All
Pou3f2 (Brn-2)	POU class 3 homeobox 2	L2–4	All
Pou3f3 (Brn-1)	POU class 3 homeobox 3	L2–4	All
Ppp1r1b^*^	Protein phosphatase 1, regulatory (inhibitor) subunit 1B	L6	Rostral
Ptn	Pleiotrophin	CPN	All
Ptprz1	Protein tyrosine phosphatase, receptor-type, Z polypeptide 1	L2–3	Caudal
Pvrl3	Poliovirus receptor-related 3	L2–3, CPN	Caudomedial
Reln^*^	Reelin	Cajal–Retzius	All
Rgs8	Regulator of G-protein signaling 8	L2–3	All
Rorb^*^	RAR-related orphan receptor B	L4	Rostral
S100a10	S100 calcium binding protein A10	L5 corticospinal motor neuron	All
Satb2	SATB homeobox 2	CPN	All
Sorl1	Sortilin-related receptor, L(DLR class) A repeats containing	L2–3	All
Sox5^*^	SRY (sex determining region Y)-box 5	L6	All
Tle1	Transducin-like enhancer of split 1 (E(sp1) homolog, Drosophila)	L2–3	All
Tle4^*^	Transducin-like enhancer of split 4	L6	All
Tox	Thymocyte selection-associated high mobility group box	L5	Rostromedial
Trp73	Tumor protein p73	Cajal–Retzius	All
Unc5c	Unc-5 homolog C (C. elegans)	L4	All
Unc5d (Svet1)	Unc-5 homolog D (C. elegans)	L2–4	All
Wnt7b^*^	Wingless-type MMTV integration site family, member 7B	L6	All
Wscd1	WSC domain containing 1	L6	Rostral
Zfpm2 (FOG2)	Zinc finger protein, FOG family member 2	L6	All

* *Genes also identified in our microarray analysis (Huang et al., 2014). CPN, callosal projection neurons; SCPN, subcerebral projection neurons*.

In our laboratory, we have identified more than 124 genes that are regulated by TBR1 at E16.5 (Huang et al., 2014; Chuang et al., 2015). The 16 region- or layer-specific genes presented in work by Bedogni et al. (2010) from Dr. Hevner's laboratory are also included in our gene list (Table 2). Moreover, based on a literature search and the database of the Simons Foundation Autism Research Initiative (https://gene.sfari.org/autdb/Welcome.do), 23 ASD-associated genes and a dyslexia causative gene, Kiaa0319, have also been found to be regulated by TBR1. Changes in the expression of these ASD- and dyslexia-associated genes (Table 3) provide support for the influence of TBR1 in ASDs. Tbr1 might act as master gene controlling the expression of a panel of ASD-associated genes and thus influence neural development and function (Chuang et al., 2015).

**Table 3:** **TBR1 influences the expression (upregulation or downregulation) of genes associated with autism or dyslexia**.

**Gene symbol**	**Gene name**	**Molecular function**	**Tbr1^−∕−^ vs. WT**	**Disease**
Cd44	CD44 molecule (Indian blood group)	Cell adhesion	Up	Autism
Cdh8	Cadherin 8, type 2	Cell adhesion	Up	Autism
Cntn6	Contactin 6	Cell adhesion	Up	Autism
Gpc6	Glypican 6	Cell adhesion	Up	Autism
Ntng1	Netrin G1	Cell adhesion	Up	Autism
Kiaa0319	Hypothetical protein D130043K22	Cell adhesion	Up	Dyslexia
Nefl	Neurofilament, light polypeptide	Cytoskeleton	Up	Autism
Gpd2	Glycerol-3-phosphate dehydrogenase 2 (mitochondrial)	Enzyme	Up	Autism
Drd1	Dopamine receptor D1A	Neurotransmission	Down	Autism
Gad1	Glutamate decarboxylase 1 (brain, 67 kDa)	Neurotransmission	Up	Autism
Grin2b	Glutamate receptor, ionotropic, N-methyl D-aspartate 2B	Neurotransmission	Down	Autism
Baiap2	BAI1-associated protein 2	Signaling	Down	Autism
Lasp1	LIM and SH3 protein 1	Signaling	Down	Autism
Lypd6	LY6/PLAUR domain containing 6	Signaling	Down	Autism
Ppp1r1b	Protein phosphatase 1, regulatory (inhibitor) subunit 1B	Signaling	Down	Autism
Ptprk	Protein tyrosine phosphatase, receptor type, K	Signaling	Down	Autism
Reln	Reelin	Signaling	Down	Autism
Auts2	Autism susceptibility candidate 2	Transcription factor	Down	Autism
Nfia	Nuclear factor I/A	Transcription factor	Down	Autism
Nr4a2	Nuclear receptor subfamily 4, group A, member 2	Transcription factor	Down	Autism
Sox5	SRY (sex determining region Y)-box 5	Transcription factor	Down	Autism
Slc4a10	Solute carrier family 4, sodium bicarbonate transporter, member 10	Transporter	Up	Autism
Stxbp6	Syntaxin binding protein 6 (amisyn)	Vesicle trafficking	Up	Autism
Sv2b	Synaptic vesicle glycoprotein 2B	Vesicle trafficking	Down	Autism

Moreover, the expression levels of 15 transcriptional regulators are reduced, while those of three transcription factors are upregulated in *Tbr1*^−∕−^ brains compared with wild-type littermates (Table 4). These changes suggest that, in addition to directly regulating gene expression, TBR1 also controls transcriptional networks to influence neuronal development. Indeed, evidence has indicated that TBR1 directly binds to the locus of *Fezf2*, a layer 5-specific transcription factor, and represses *Fezf2* expression in layer 6 to specify the corticothalamal projections of layer 6 neurons (Han et al., 2011; McKenna et al., 2011). The second transcriptional regulator directly controlled by TBR1 is autistic susceptibility gene2 (*Auts2*). TBR1 binds to the region around the *Auts2* transcriptional start site and activates expression of the *Auts2* gene (Bedogni et al., 2010). AUTS2 is part of polycomb repressive complex I (PRCI) that catalyzes the monoubiquitination of histone H2A and epigenetically represses gene expression, particularly during the developmental stage (de Napoles et al., 2004; Wang et al., 2004b). In contrast to the canonical role of PRCI in gene repression, the PRCI–AUST2 complex activates neuronal gene expression by recruiting casein kinase 2 and p300 to chromatin (Gao et al., 2014). The activation of *Auts2* expression by TBR1 supports the influence of TBR1 on global gene expression in neurons.

**Table 4:** **Deletion of ***Tbr1*** influences the expression of a panel of transcription factors in neurons**.

**Gene Symbol**	**Tbr1^−∕−^ vs. WT**	**Gene name**
Bcl6	0.3623	B-cell leukemia/lymphoma 6
Nfe2l3	0.4017	Nuclear factor, erythroid derived 2, like 3
Nhlh2	0.4219	Nescient helix loop helix 2
Sox5	0.4875	BB018032 RIKEN full-length enriched, adult male testis (DH10B) *Mus musculus* cDNA clone 4930572C18 3′ similar to AJ010604 *Mus musculus* mRNA for transcription factor L-Sox5, mRNA sequence.
Nr4a2	0.5179	BB703394 RIKEN full-length enriched, *in vitro* fertilized eggs *Mus musculus* cDNA clone 7420451N07 3′, mRNA sequence.
Rorb	0.529	RAR-related orphan receptor beta
Tp73	0.5355	Transformation related protein 73
Btbd11	0.5719	BTB (POZ) domain containing 11
Tle4	0.5821	Transducin-like enhancer of split 4, homolog of Drosophila E(spl)
Rbm14	0.59	RNA binding motif protein 14
Foxf2	0.5983	Forkhead box F2
Tfap2c	0.6348	Transcription factor AP-2, gamma
Zswim4	0.639	Zinc finger, SWIM domain containing 4
Nfia	0.643	RIKEN cDNA 9430022M17 gene
Neurod6	0.6644	Neurogenic differentiation 6
Runx1t1	1.5064	CBFA2T1 identified gene homolog (human)
Ascl1	1.6328	Achaete–scute complex homolog-like 1 (Drosophila)
Pou3f1	1.6528	POU domain, class 3, transcription factor 1

In addition to using a transcriptional cascade to indirectly control gene expression, TBR1 may also alter the relative number of projection neurons and interneurons in the brain and influence the total expression levels of certain genes, such as *Gad1*, which encodes glutamate decarboxylase 1 (GAD67)—an essential gene of GABAergic neurons. In *Tbr1* deletion mice, the expression of *Gad1* is noticeably upregulated (Chuang et al., 2015). Because Tbr1 is specifically expressed in glutamatergic projection neurons, it is possible to speculate that increased *Gad1* expression is indirectly linked to a reduction in the population of glutamatergic neurons.

Consistent with the function of TBR1 in the regulation of axonal projection, TBR1 also regulates eight membrane proteins (CNTN2, CDH8, GPC6, CD44, FLRT3, CNTN6, NTNG1, and KIAA0319) that are involved in cell adhesion; although it is still unclear whether these genes are directly or indirectly regulated by TBR1 (Chuang et al., 2015). Interestingly, seven of these eight membrane proteins are upregulated in *Tbr1*^−∕−^ brains (Table 5). Because these genes control cell adhesion and axonal growth, the impairment of axonal projection in *Tbr1* deficient neurons is likely due to imbalanced cell–cell and cell–matrix interactions. Alteration of the strength of these interactions may preclude neurite growth and extension (Chuang et al., 2015).

**Table 5:** *****Tbr1*** deletion mainly upregulates expression of cell adhesion molecules in neurons**.

**Gene symbol**	**Tbr1^−∕−^ vs. WT**	**Gene name**
Cdh8	1.5013	Cadherin 8
Gpc6	1.5874	Glypican 6
Cd44	1.7384	CD44 antigen
Flrt3	1.8118	Fibronectin leucine rich transmembrane protein 3
Cntn6	2.0967	Contactin 6
Ntng1	2.3218	Netrin G1
Kiaa0319	2.8456	Hypothetical protein D130043K22
Cntn2	0.6331	Contactin 2

In conclusion, TBR1 controls the expression of a series of genes that regulate cell-cell adhesion, axonal growth, neurotransmission and gene expression.

## TBR1 interacting proteins

To date, only two proteins, CASK and FOXP2, have been identified as interacting partners with TBR1. Both CASK and FOXP2 are associated with ASDs (Samuels et al., 2007; O'roak et al., 2011). TBR1 was identified as a binding partner for CASK from a yeast two-hybrid screen using the guanylate kinase domain of CASK as bait (Hsueh et al., 2000). The C-terminal region of TBR1 is required for the interaction with CASK (Hsueh et al., 2000). CASK—a multidomain adaptor protein—is widely distributed in various subcellular compartments and interacts with more than two dozen cellular proteins (Hsueh, 2006). The interaction with CASK increases the transcriptional activity of TBR1 (Hsueh et al., 2000) by recruiting a nucleosome assembly protein CINAP (CASK interacting nucleosome assembly protein, also known as testis specific protein Y-encoded like 2, TSPYL2) to the promoter region containing the T-box DNA binding motif (Wang et al., 2004a). CINAP also interacts with the guanylate kinase domain of CASK. However, it does not compete with TBR1 for CASK binding. Instead, TBR1, CASK and CINAP form a tripartite complex to regulate *Grin2b* expression (Wang et al., 2004a,c). *CASK* is well-known as a causative gene in X-linked mental retardation (Najm et al., 2008). The interaction of CASK and TBR1 and the consequent effect on the regulation of *Grin2b* expression and neural development has been suggested to contribute to the phenotype of patients with *CASK* mutations (Hsueh, 2009). It can also be speculated that *Grin2b* expression, as controlled by the TBR1–CASK complex, might also be involved in ASDs due to *TBR1* or *CASK* mutations.

FOXP2 is a critical transcription factor that controls speech (Lai et al., 2001; Enard et al., 2002) and is also associated with ASDs (Gong et al., 2004; Li et al., 2005). In contrast to the interaction between CASK and TBR1, the interaction between TBR1 and FOXP2 is less clear. Research suggests that both the T-box and C-terminal regions of TBR1 are involved in the interaction with FOXP2 (Deriziotis et al., 2014). For FOXP2, both its N- and C-terminal regions contribute to the interaction between FOXP2 and TBR1 (Deriziotis et al., 2014). Although it has been speculated that the interaction of FOXP2 and TBR1 is likely relevant to the verbal deficits in ASD patients, the molecular function of the TBR1-FOXP2 interaction is unclear. Furthermore, FOXP2 and TBR1 are only coexpressed in layer 6 of the cerebral cortex and not in other layers of the cerebral cortex and amygdala. Thus, the interaction with FOXP2 can only partly account for the function of TBR1.

TBR1, its binding partners CASK and FOXP2 and its direct downstream target GRIN2B, are all associated with ASDs, reinforcing the role of TBR1 in ASDs.

## TBR1 mutations associate with neurological disorders

Genetic analyses of patients have identified *TBR1* as a high-confidence risk factor for ASDs (https://gene.sfari.org/autdb/GSGeneList.do?c=1). Identified mutations in TBR1 genes are summarized in Figure 1. Both *de novo* and inherited mutations in *TBR1* have been found in patients with ASDs (Figure 1). Two of the mutations, p.A136PfsX80 and p.S351X, result in early termination and generate truncated proteins that lack a functional DNA-binding T-box domain (O'roak et al., 2012a,b; De Rubeis et al., 2014). These two truncated mutants can no longer function in transcription or in interactions with CASK and FOXP2. The remaining three *de novo* mutations are p.K228E, p.W271C, and p.N374H (Figure 1). Based on simulations with the T-box DNA binding domain of Brachury (http://www.ncbi.nlm.nih.gov/Structure/cdd/cddsrv.cgi?uid=250216), the K228 residue is predicted to directly contribute to DNA binding (Figure 1). Thus, the p.K228E mutation is expected to disrupt the DNA binding ability of TBR1. The residues W271 and N374 are adjacent to the DNA binding residues (Figure 1). Thus, the p.W271C and p.N374H mutations could alter protein conformation and indirectly influence DNA binding. The p.V356M inherited mutation is localized in the T-box, but it is relatively far from the DNA binding and dimerization motifs. The remaining inherited mutations, including p.E31Q, p.P38T, p.Q178E, p.Q418R, p.V437L, and p.P542R, are localized to the N- and C-terminal regions and are not known to influence DNA binding. The impact of these inherited mutations is unclear.

To date, only two studies have analyzed the effects of these ASD mutations on TBR1 function. We contributed to the first functional study, which examined axonal growth in the amygdalar neurons of the TBR1 N374H mutant (Huang et al., 2014). An experiment comparing *Tbr1*^+∕−^ and wild-type amygdalar neurons showed that the deletion of one *Tbr1* gene results in multiple and shorter axons in amygdalar neurons (Huang et al., 2014). The reintroduction of wild-type *Tbr1* into *Tbr1*^+∕−^ amygdalar neurons effectively promotes axon growth and reduces the percentage of neurons carrying multiple axons to the levels seen in wild-type neurons. However, the N374H mutant fails to rescue the axonal defects of *Tbr1*^+∕−^ amygdalar neurons, suggesting that the p.N374H mutation identified in patients with ASD results in a loss of function (Huang et al., 2014).

The second study focused on the effect of ASD mutations on the subcellular distribution, transcriptional activity, dimerization and protein-protein interaction of TBR1 using heterologous HEK293 cells as a model (Deriziotis et al., 2014). De novo mutations, including p.K228E and p.N374H, change the subcellular distribution of TBR1 in HEK293 cells. The mutant proteins tend to form large aggregates in the nuclei. The impact of these two mutations on the transcriptional activity of TBR1 is unclear because the luciferase reporter assay did not show a difference between the wild-type TBR1 and K228E and N374H mutants. However, similar to the truncated mutants, the K228E and N374H mutants no longer interact with FOXP2, which is consistent with the observation that the T-box domain is also involved in FOXP2 interactions, as described above. The mechanisms by which rare inherited mutations impair the function of TBR1 remain largely unclear, except for the p.Q418R mutation, which is known to reduce the interaction between TBR1 and FOXP2. Because TBR1 is a projection neuron-specific transcription factor, the relevance of the interaction between TBR1 and FOXP2 must be investigated in neurons instead of HEK293 cells.

In addition to ASDs, *TBR1* is also associated with intellectual disability. The *TBR1* locus is at chromosome 2q24.2. Both a microdeletion of the chromosome region that contains 2q24.2 and *de novo* mutations of the *TBR1* gene have been found in patients with intellectual disabilities (Traylor et al., 2012; Burrage et al., 2013; Hamdan et al., 2014; Palumbo et al., 2014). Moreover, the expression levels of TBR1 are increased in patients that suffer from schizophrenia (Molnar et al., 2003). Taken together, TBR1 is closely associated with ASDs, schizophrenia and intellectual disability.

## *Tbr1* haploinsufficiency results in neuronal defects

In ASD patients, only one of the two *TBR1* alleles is mutated (Neale et al., 2012; O'roak et al., 2012a,b; De Rubeis et al., 2014; Deriziotis et al., 2014). Several possibilities may explain the effect of *TBR1* heterozygosity on brain function: haploinsufficiency or a dominant negative or gain-of-function effect of the mutated allele. Because two of the *de novo* mutations of the *TBR1* gene, p.A136PfsX80, and p.S351X, result in early termination and generate truncated proteins that lack a full length T-box DNA binding domain and dimerization domain (Figure 1), the mutants are not expected to exert a dominant negative effect on the activity of TBR1 based on the known molecular function of TBR1. Instead, the defects are likely caused by haploinsufficiency. *Tbr1*^+∕−^ neurons are characterized by shorter and multiple axons (Huang et al., 2014), indicating that loss of a copy of the *Tbr1* gene results in abnormal neuronal differentiation. Thus, *TBR1* deficits in patients are likely due to haploinsufficiency.

## *Tbr1^+∕−^* mice serve as a mouse model for ASDs

Because only one of two *TBR1* alleles is mutated in patients with ASDs and *Tbr1* heterozygosity does not influence survival and the general health of mice, *Tbr1*^+∕−^ mice serve as a good animal model to elucidate the role of *Tbr1* in ASDs. The core symptoms of patients with ASDs are both verbal and non-verbal communication defects, impaired social interaction and cognitive inflexibility. ASDs are also frequently associated with learning disability. A series of behavior paradigms have been applied to characterize the behavioral defects of *Tbr1* (Table 6). Compared with wild-type littermates, the locomotor and exploratory activities, the level of anxiety and the hippocampus-dependent memory of *Tbr1*^+∕−^ mice are normal (Huang et al., 2014). However, the amygdala-dependent behaviors of *Tbr1*^+∕−^ mice are noticeably affected. Conditioned taste aversion and auditory fear conditioning—two amygdala-dependent learning and memory paradigms—are both impaired in *Tbr1*^+∕−^ mice. Cognitive flexibility, as examined by appetitive-motivated T-maze and two-choice digging tests, is also noticeably reduced in *Tbr1*^+∕−^ mice. The three-chamber test, reciprocal social interactions and social transmission of food preferences have also been applied to characterize the social interactions of *Tbr1*^+∕−^ mice. These paradigms all indicate that the social interactions of *Tbr1*^+∕−^ mice are impaired. Moreover, the frequency of ultrasonic vocalization is significantly lower in isolated *Tbr1*^+∕−^ pups. Thus, these behavioral analyses strongly support that *Tbr1*^+∕−^ mice exhibit autism-like behaviors (Huang et al., 2014).

**Table 6:** *****Tbr1*** haploinsufficiency results in autism-like behaviors in mice**.

**Behavior paradigm**	**Assay for**	**Phenotype in Tbr1^+∕−^ mice**
Conditioned taste aversion	Learning and memory	Learning defect
Auditory fear conditioning	Learning and memory	Learning defect
T-maze test	Reverse learning	Cognition inflexibility
Two-choice digging task	Reverse learning	Cognition inflexibility
Three-chamber test	Social interaction	Poor social interaction
Reciprocal social interactions	Social interaction	Poor social interaction
Social transmission of food preference	Social interaction	Poor social interaction
Ultrasonic vocalizations	Communication	Poor communication

## Defects in amygdalar circuits and activation are critical for ASDs caused by *Tbr1* deficiency

When *Tbr1* is completely deleted from mice, the most prominent phenotypes are observed in the neuronal migration and axonal projection of the embryonic cerebral cortex (Bulfone et al., 1998; Hevner et al., 2001). However, none of these defects are found in the *Tbr1*^+∕−^ brain (Huang et al., 2014). Cortical lamination, contralateral cortical projection, corticothalamal projection and the size of the cerebral cortex of *Tbr1*^+∕−^ brains are comparable to those of wild-type brains (Huang et al., 2014). Unexpectedly, the posterior part of the anterior commissure is either missing or dramatically reduced in *Tbr1*^+∕−^ mice (Huang et al., 2014). This defect is 100% penetrant in all *Tbr1*^+∕−^ mice. Thus, the posterior part of the anterior commissure is the structure most sensitive to *Tbr1* haploinsufficiency. Consequently, defects of the posterior part of the anterior commissure are more relevant to the pathogenesis of *TBR1*-dependent ASDs.

**Figure 2:**
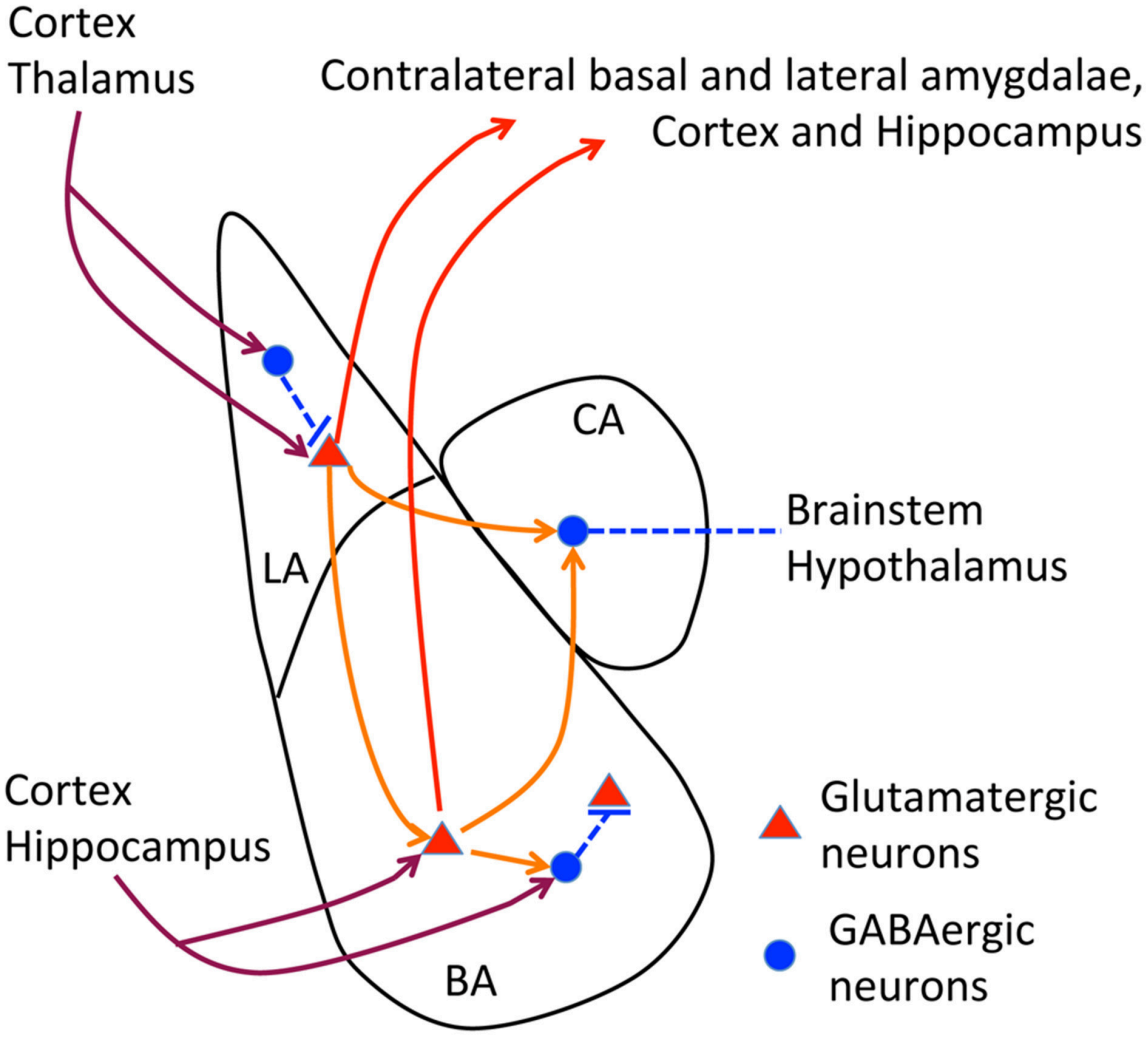
**Neural circuits of amygdala**. The relative position of lateral, basal and central amygdalae and the known axonal connections of amygdala are summarized. In lateral and basal amygdalae, glutamatergic neurons comprise the majority of cells. In contrast, GABAergic neurons are the major population in the central amygdala. Dark red lines, afferent pathways from cortex, thalamus and hippocampus; red lines, efferent pathways to contralateral amygdala, cortex and hippocampus; orange lines, intraamygdalar pathways. The central amygdala also delivers signals to the brainstem and hypothalamus to regulate freezing and emotion responses.

The posterior part of the anterior commissure serves to connect the contralateral amygdalae (interamygdalar projections). The amygdala contains three major nuclei, namely the lateral, basal and central amygdala (Figure 2). Interamygdalar projections emerge from the lateral and basal amygdala. These two nuclei also project to the ipsilateral central amygdala (intraamygdalar projections). The lateral and basal amygdalae are the nuclei that receive inputs from the cortex, thalamus and hippocampus. To induce a freezing response, the lateral and basal amygdalae deliver the signals to the central amygdala, and the central amygdala further projects to the brainstem and hypothalamus. In addition to the central amygdala, the lateral and basal amygdalae also project back to the cortex, hippocampus, and thalamus, which are believed to regulate memory and social behavior (Lee et al., 2013; Janak and Tye, 2015). Because the amygdala is the pivotal brain structure for social intelligence, the amygdala is an obvious target for the etiology of *TBR1*-dependent ASDs.

**Figure 3:**
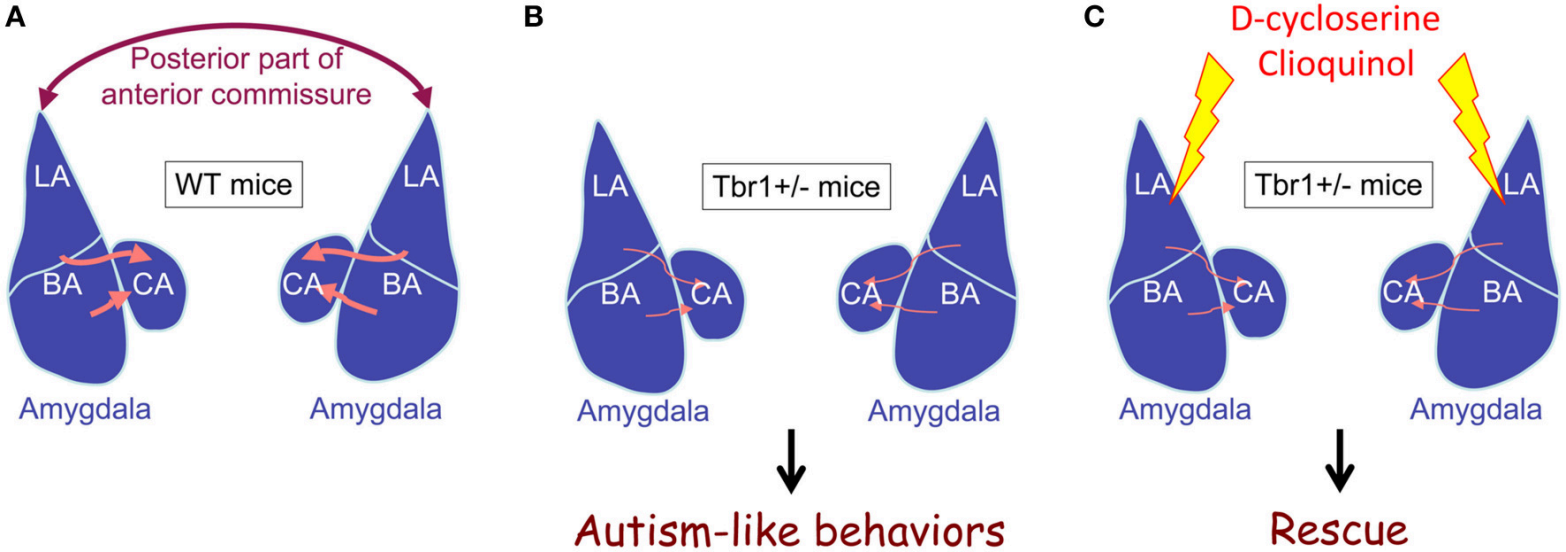
*****Tbr1*** haploinsufficiency results in defects of intra- and inter-amygdalar axonal projections**. **(A)** Wild-type mice. **(B)** *Tbr1*^+∕−^ mice. **(C)** *Tbr1*^+∕−^ mice with D-cycloserine or clioquinol injection. The interamygdalar projection via the posterior part of the anterior commissure is significantly impaired in *Tbr1*^+∕−^ mice. Intraamygdalar connections between basolateral and central amygdalae are also noticeably reduced in *Tbr1*^+∕−^ mice. *Tbr1*^+∕−^ mice are characterized by autism-like behaviors. Increased neuronal activity in the amygdala using either D-cycloserine or clioquinol restores behavioral defects to normal levels, although axonal projection defects are not rescued.

The results of DiI tracing and retrograde red bead labeling show that both inter- and intra-amygdalar axonal projections are noticeably impaired in *Tbr1*^+∕−^ mice (Figure 3; Huang et al., 2014). The lateral and basal amygdala neurons are the major target of Tbr1 haploinsufficiency. Consistent with the axonal projection defects in the brain, *Tbr1*^+∕−^ amygdala neurons possess shorter and multiple axons, which suggests that *Tbr1* haploinsufficiency results in a cell-autonomous effect that restricts axonal extension and differentiation (Huang et al., 2014). To investigate how TBR1 controls axonal growth, we examined the TBR1 downstream target genes in a gene list from our microarray data. Specifically, we examined *Cntn2, Cdh8*, and *Ntng1* because these three genes encode adhesion proteins that regulate neurite outgrowth and fasciculation (Furley et al., 1990; Stoeckli and Landmesser, 1995; Kunz et al., 1998; Nakashiba et al., 2002; Bekirov et al., 2008). Restoring the expression levels of *Cntn2, Cdh8*, and *Ntng1* in *Tbr1*^+∕−^ amygdalar neurons effectively ameliorates axonal growth and differentiation in cultures and promotes axonal projection to form the posterior part of the anterior commissure *in vivo*. Thus, TBR1 controls the expression of a panel of genes that regulate amygdalar axonal projections. Note that although the axonal projections of lateral and basal amygdalae are significantly impaired in *Tbr1*^+∕−^ brains, the size and cell density of the lateral and basal amygdalae do not differ between *Tbr1*^+∕−^ mice and wild-type littermates. It is unclear whether *Tbr1*^+∕−^ amygdalar neurons mistarget to other brain regions. More investigations are needed to characterize this regulation in detail.

Although the significance of interamygdalar connections is unclear, a reduction of intraamygdalar axonal projections implies that the amygdala is functionally impaired in *Tbr1*^+∕−^ mice. Indeed, neuronal activation of the amygdala is impaired in *Tbr1*^+∕−^ mice (Huang et al., 2014). Two amygdala-dependent learning/memory paradigms—conditioned taste aversion and auditory fear conditioning—have been used to investigate amygdalar responses. Both c-FOS and NMDAR2B protein levels were used to monitor neuronal activation in amygdalae. These experiments showed that the induction of both c-FOS and NMDAR2B are either much lower or completely absent in the lateral and basal amygdalae of *Tbr1*^+∕−^ mice after conditioned taste aversion training and auditory fear conditioning (Huang et al., 2014; Chuang et al., 2015). Thus, both axonal projection and neuronal activation are defective in *Tbr1*^+∕−^ amygdalae.

Possibly due to an impairment of NMDAR induction in the *Tbr1*^+∕−^ amygdala, electrophysiological recording data showed that the NMDA/AMPA ratio is noticeably lower in the thalamic-lateral amygdala synapses of *Tbr1*^+∕−^ brains compared with those of wild-type brains (Lee et al., 2015b). Consistent with amygdala-specific defects in *Tbr1*^+∕−^ mice, the hippocampal Schaffer collateral-CA1 pyramidal synapses do not exhibit this abnormal NMDA/AMPA ratio (Lee et al., 2015b). These electrophysiological studies clearly demonstrate a functional NMDAR deficiency in *Tbr1*^+∕−^ amygdalae.

If impairment of amygdala activation, and particularly reduced NMDAR activity, is critical for autism-like behaviors in *Tbr1*^+∕−^ mice, the activation of amygdalar neurons should ameliorate the behavioral defects of *Tbr1*^+∕−^ mice. Indeed, a bilateral local infusion of D-cycloserine (a coagonist of NMDAR) into the amygdala clearly ameliorates the reciprocal social interaction and conditioned taste aversion defects seen in *Tbr1*^+∕−^ mice (Huang et al., 2014). D-cycloserine applied 30 min before the behavioral assay does not influence the expression of TBR1 target genes, suggesting the behavioral effects are mediated by an acute enhancement of NMDAR transmission. Because administration of D-cycloserine to the amygdala is sufficient to ameliorate the behavioral defects of *Tbr1*^+∕−^ mice, the etiology of autism-like behaviors in *Tbr1*^+∕−^ mice very likely involves amygdala defects (Huang et al., 2014).

In addition to local infusion, systemic administration of D-cycloserine via an intraperitoneal injection also effectively restores neuronal activation of the *Tbr1*^+∕−^ amygdala and improves social interaction, cognitive inflexibility and associative memory of *Tbr1*^+∕−^ mice (Huang et al., 2014). These results indicate a potential therapeutic avenue for ASD patients possessing *TBR1* gene mutations. *Tbr1*^+∕−^ mice are not the only mouse model to have been used to demonstrate the beneficial effect of D-cycloserine. The behavioral deficits of *Shank2*^−∕−^, *Nlgn1*^−∕−^, and *Grid1*^−∕−^ mice can also be ameliorated by systemic administration of D-cycloserine. Specifically, D-cycloserine improves social interactions in both *Shank2*^−∕−^ and *Grid1*^−∕−^ mice in a three-chamber test (Won et al., 2012; Yadav et al., 2012). Furthermore, D-cycloserine reduces the repetitive grooming behavior of *Nlgn1*^−∕−^ mice (Blundell et al., 2010). These three mutant mice all show NMDAR defects, which is consistent with the idea that NMDAR deficits are critical to the etiology of ASDs (Lee et al., 2015a). Improving NMDAR activity can ameliorate the behavioral defects of these mutant mice.

To further support the NMDAR deficit hypothesis in ASDs, we recently showed that by improving NMDAR activity via the administration of clioquinol, the social defects of *Tbr1*^+∕−^ mice are rescued (Lee et al., 2015b). Clioquinol is a zinc chelator and ionophore that promotes the mobilization of zinc from presynaptic vesicles to the postsynaptic site. The postsynaptic elevation of zinc activates the protein tyrosine kinase SRC and consequently enhances NMDAR activity. Systemic administration of clioquinol noticeably improves the sociability of the mutant mice in the three-chamber test. Consistent with the behavioral rescue, the defective electrophysiological responses of mutant brains are also ameliorated by clioquinol treatment. In *Tbr1*^+∕−^ brains, clioquinol can restore the reduced NMDA/AMPA ratio of the thalamic-lateral amygdala synapses. Clioquinol treatment also shows a beneficial effect on *Shank2*^−∕−^ mice. It enhances the NMDAR activity of hippocampal Schaffer collateral-CA1 pyramidal synapses in *Shank2*^−∕−^ mice (Lee et al., 2015b). Even though the molecular mechanisms responsible for the NMDAR deficit differ between *Tbr1*^+∕−^ and *Shank2*^−∕−^ mice, increasing NMDAR activity via D-cycloserine or clioquinol efficiently ameliorates the behavioral defects of these two mutant mice. These data support that an NMDAR deficit is likely to be a common pathogenic mechanism of ASDs. Moreover, studies using D-cycloserine and clioquinol suggest that activation of amygdalar neurons using suitable pharmacological treatments can ameliorate the behavioral defects caused by *Tbr1* haploinsufficiency, even though the axonal projection defects of the *Tbr1*^+∕−^ amygdala cannot be rescued in adult animals.

## *Tbr1* serves as immediate early gene to control neuronal activation in mature neurons

Although the expression levels of *Tbr1* gradually decline after birth, the protein levels of TBR1 remain detectable in adult mouse brains (Hsueh et al., 2000; Hong and Hsueh, 2007). Based on the following scenario, TBR1 may also play a role in the adult brain. TBR1 regulates *Grin2b* expression (Wang et al., 2004a), and CASK phosphorylation by protein kinase A (PKA) enhances this regulation. CASK phosphorylation increases the interaction between TBR1 and CASK and thus upregulates *Grin2b* promoter activity (Huang et al., 2010). Therefore, PKA phosphorylation may increase the ability of TBR1 to influence *Grin2b* expression, even though the expression levels of *Tbr1* are lower in adult brains.

**Figure 4:**
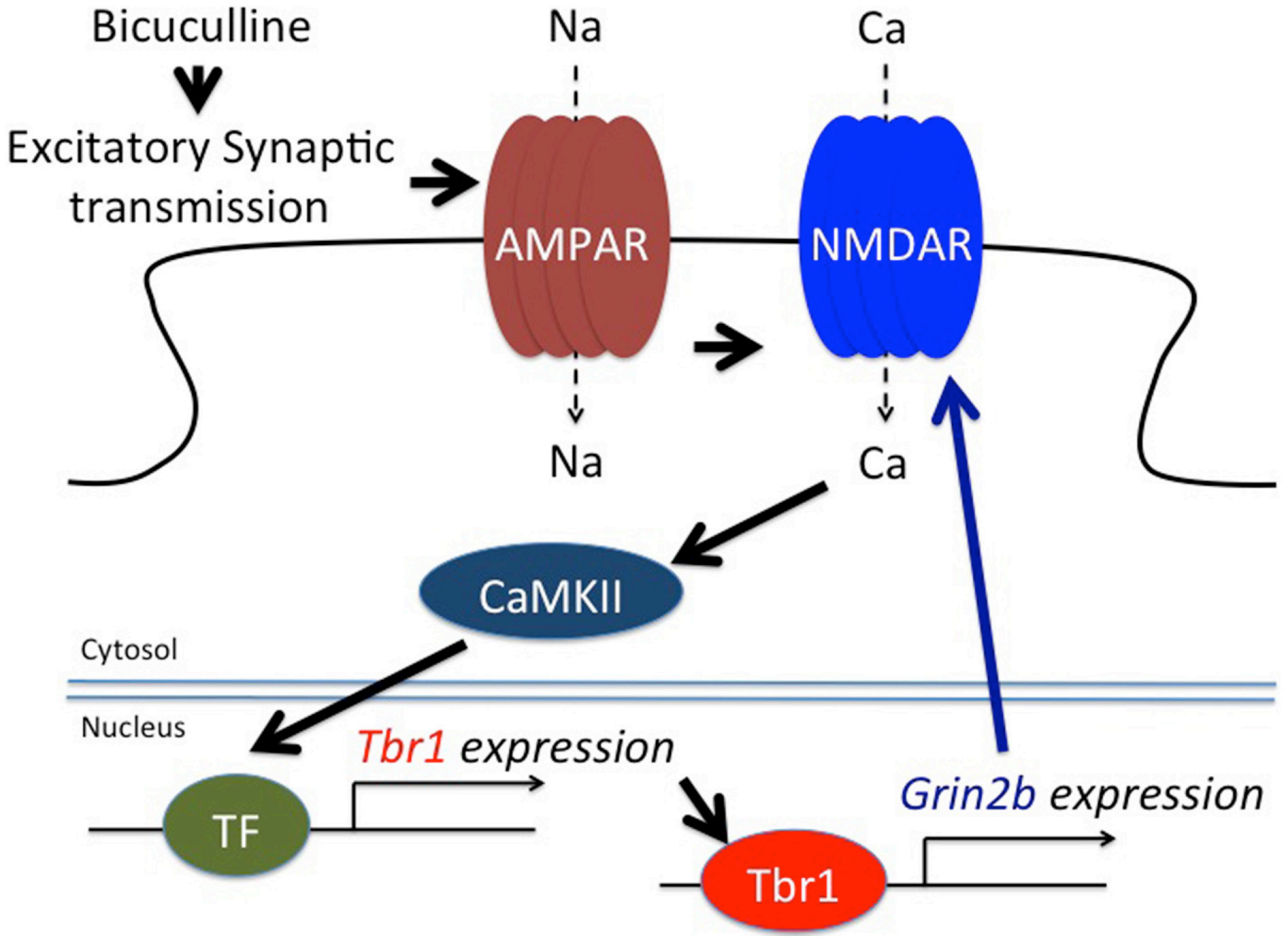
**TBR1 acts as an immediate early gene to induce ***Grin2b*** expression during neuronal activation**. Both bicuculline, an antagonist of GABA_A_ receptors, and glutamate treatment induce *Tbr1* expression. Although the transcription factor (TF) required for *Tbr1* expression is unknown, evidence indicates that CaMKII is involved in *Tbr1* upregulation. TBR1 can then induce *Grin2b* expression to enhance the synaptic responses (adapted from Chuang et al., 2014).

A study of *in vitro* cultured neurons unexpectedly showed that glutamate and bicuculline treatments noticeably upregulates *Tbr1* expression (Chuang et al., 2014). Two to six hours after glutamate or bicuculline treatment, both the RNA and protein levels of *Tbr1* are obviously increased. This induction is transient. The RNA levels of *Tbr1* are decreased to basal levels 12 h after stimulation. This feature is shared among the cortical, hippocampal, and amygdalar neurons, although the induction of *Tbr1* expression is much less pronounced in hippocampal neurons (Chuang et al., 2014). In addition to cultured neurons, behavioral stimulation also changes *Tbr1* expression levels in mouse brains. When conditioned taste aversion is applied to stimulate neuronal activation, similar to *c-Fos* induction, *Tbr1* RNA levels in the lateral amygdala, the insular cortex and the ventral hippocampus are also transiently increased 2 h after training (Chuang et al., 2014). Neuronal activation also induces *Grin2b* expression *in vitro* and *in vivo*, but this induction occurs several hours after that of *Tbr1*. Moreover, deletion of *Tbr1* completely blocks *Grin2b* induction in culture (Chuang et al., 2014). Both NMDAR and CaMKII are required to induce *Tbr1* expression (Figure 4). Thus, in addition to regulating axonal differentiation and neuronal migration during the early developmental stage, *Tbr1* also acts as an immediate early gene in response to synaptic stimulation in mature neurons, which might contribute to the etiology of *TBR1*-related ASDs. In particular, the cerebral cortex of *Tbr1*^+∕−^ mice likely exhibits defective electrophysiological responses and thus influences behaviors, even though anatomic defects of the cerebral cortex have not been identified in *Tbr1*^+∕−^ mice. More investigations need to be conducted to address this possibility.

## Abnormal brain wiring and excitation/inhibition imbalance—two prominent models for the pathogenic mechanism of ASDs

Although the etiology of ASDs is heterogeneous, the two most prominent models for autism pathogenesis in the literature are abnormal brain wiring and an imbalance of neuronal activity (excitatory/inhibitory imbalance; Rubenstein and Merzenich, 2003; Walsh et al., 2008; Bernardinelli et al., 2014; Cellot and Cherubini, 2014; Deidda et al., 2014). These two defects lead to abnormal information processing and result in autism-like behaviors. These two models are interconnected to a certain extent. During neurodevelopment, neurons must extend their axons and form synapses with their target neurons, which allows the activity of the downstream target neurons to be regulated. In the absence of correct excitatory or inhibitory inputs, the activity of target neurons will be either too low or too high. These inappropriate levels of neuronal activity result in abnormal information processing, which leads to aberrant behaviors. Moreover, the imbalanced activity of neurons also influences (either strengthens or attenuates) their connections to other neurons. When the connection is too weak, it may be eliminated, which may alter brain wiring. In the *Tbr1*^+∕−^ mouse model, amygdalar axonal projections are defective. Both inter- and intra-amygdalar connections are noticeably impaired. Moreover, the NMDAR activity of amygdalar neurons is also much lower in *Tbr1*^+∕−^ brains. Thus, *Tbr1*^+∕−^ brains are characterized by both abnormal brain wiring and defective neuronal activation. Further investigation is required to see whether one deficit contributes more substantially to ASD pathology.

## Concluding remarks

*Tbr1*^+∕−^ mice constitute the first genetic mouse model to show that defects in amygdalar circuits and activity result in autism-like behaviors. TBR1 controls the expression of a panel of genes that is associated with ASDs. TBR1 regulates axonal growth and the neuronal activation of amygdalar neurons by regulating downstream genes. Enhancing NMDAR activation with D-cycloserine and clioquinol to increase neuronal activity can ameliorate the behavioral defects of *Tbr1*^+∕−^ mice, although the anatomic defects caused by *Tbr1* haploinsufficiency are not rescued. *Tbr1*^+∕−^ mice thereby serve as a model to elucidate how mutation of an autism causative gene influences brain wiring and impairs neuronal activity and consequently results in autism-like behaviors. Nevertheless, several issues remain unresolved. First, why is the amygdala the brain structure most sensitive to *Tbr1* haploinsufficiency? Second, since *Tbr1*^+∕−^ amygdalar neurons do not correctly form inter- and intra-amygdalar connections, a study of the mistargeting of *Tbr1*^+∕−^ amygdalar axons might further illustrate the neural circuit defects caused by *Tbr1* haploinsufficiency. Third, although the anatomical features of the cerebral cortex of *Tbr1*^+∕−^ mice are normal, the electrophysiological responses of the *Tbr1*^+∕−^ cerebral cortex remain to be measured. Fourth, the TBR1 downstream genes have not been annotated in detail. Only four TBR1 direct target genes have so far been identified. Further work is necessary to understand the individual actions of other TBR1 downstream target genes and how their dysfunction could generate autism-like behaviors. Finally, it is unclear how TBR1 functions as a transcriptional activator in some cases but acts as a repressor in others. This phenomenon also deserves further investigation. Addressing these questions will further elucidate the roles of TBR1 in brains and potentially impact on autism research.

## Author contributions

Both YH and TH wrote the manuscript and prepared the tables and figures.

### Conflict of interest statement

The authors declare that the research was conducted in the absence of any commercial or financial relationships that could be construed as a potential conflict of interest.
